# Analysis of WSI Images by Hybrid Systems with Fusion Features for Early Diagnosis of Cervical Cancer

**DOI:** 10.3390/diagnostics13152538

**Published:** 2023-07-31

**Authors:** Mohammed Hamdi, Ebrahim Mohammed Senan, Bakri Awaji, Fekry Olayah, Mukti E. Jadhav, Khaled M. Alalayah

**Affiliations:** 1 Department of Computer Science, College of Computer Science and Information Systems, Najran University, Najran 66462, Saudi Arabia; balawaji@nu.edu.sa; 2 Department of Artificial Intelligence, Faculty of Computer Science and Information Technology, Alrazi University, Sana’a, Yemen; 3 Department of Information Systems, College of Computer Science and Information Systems, Najran University, Najran 66462, Saudi Arabia; Dr.fekry_olayah@yahoo.com; 4 Shri Shivaji Science & Arts College, Chikhli Dist., Buldana 443112, India; muktijadhav@gmail.com; 5 Department of Computer Science, College of Science and Arts, Najran University, Sharurah 68341, Saudi Arabia; kmalalayah@nu.edu.sa

**Keywords:** deep learning, RF, SVM, fusion features, ACA, WSIs, cervical cancer

## Abstract

Cervical cancer is one of the most common types of malignant tumors in women. In addition, it causes death in the latter stages. Squamous cell carcinoma is the most common and aggressive form of cervical cancer and must be diagnosed early before it progresses to a dangerous stage. Liquid-based cytology (LBC) swabs are best and most commonly used for cervical cancer screening and are converted from glass slides to whole-slide images (WSIs) for computer-assisted analysis. Manual diagnosis by microscopes is limited and prone to manual errors, and tracking all cells is difficult. Therefore, the development of computational techniques is important as diagnosing many samples can be done automatically, quickly, and efficiently, which is beneficial for medical laboratories and medical professionals. This study aims to develop automated WSI image analysis models for early diagnosis of a cervical squamous cell dataset. Several systems have been designed to analyze WSI images and accurately distinguish cervical cancer progression. For all proposed systems, the WSI images were optimized to show the contrast of edges of the low-contrast cells. Then, the cells to be analyzed were segmented and isolated from the rest of the image using the Active Contour Algorithm (ACA). WSI images were diagnosed by a hybrid method between deep learning (ResNet50, VGG19 and GoogLeNet), Random Forest (RF), and Support Vector Machine (SVM) algorithms based on the ACA algorithm. Another hybrid method for diagnosing WSI images by RF and SVM algorithms is based on fused features of deep-learning (DL) models (ResNet50-VGG19, VGG19-GoogLeNet, and ResNet50-GoogLeNet). It is concluded from the systems’ performance that the DL models’ combined features help significantly improve the performance of the RF and SVM networks. The novelty of this research is the hybrid method that combines the features extracted from deep-learning models (ResNet50-VGG19, VGG19-GoogLeNet, and ResNet50-GoogLeNet) with RF and SVM algorithms for diagnosing WSI images. The results demonstrate that the combined features from deep-learning models significantly improve the performance of RF and SVM. The RF network with fused features of ResNet50-VGG19 achieved an AUC of 98.75%, a sensitivity of 97.4%, an accuracy of 99%, a precision of 99.6%, and a specificity of 99.2%.

## 1. Introduction

Cancer is a curse that threatens human life. It is characterized by the growth of abnormal cells, as well as their abnormal and out-of-control reproduction. Malignant cells invade and cause damage to neighboring organs. Any abnormal growth is a precursor to a tumor, whether benign or malignant. The benign tumor remains in its place and does not invade the adjacent normal tissues [1], while the malignant tumors infiltrate the neighboring normal cells and destroy them [2]. Cervical cancer is the fourth type of malignant tumor that threatens a woman’s life if detected late [3]. Cervical cancer appears in the lower cervical tissues, which grow abnormally and out of control [4]. According to World Health Organization reports [5], 342,000 women died in 2020 due to cervical cancer [6]. Human papillomavirus (HPV) is a virus of the genital tract and is the main cause of cervical cancer [7]. HIV-positive women are more likely to develop cervical cancer at a younger age and with more advanced disease. This is because HIV infection can speed up the progression of HPV infection from precancerous to cancerous cells [8]. Cervical cancer slowly grows and spreads, so no symptoms appear in the early stages [9]. Therefore, regular screening is the best way to prevent cervical cancer. Squamous cell carcinoma is a type of cancer that begins in the squamous cells, which are the flat cells that line the outer part of the cervix. It is the most common type of cervical cancer, accounting for about 80% of all cases. Squamous cell carcinomas are often aggressive and can spread quickly to other parts of the body if not diagnosed and treated early [10]. There are some symptoms that occur in the advanced stages, such as abnormal vaginal bleeding, hematuria, pelvic pain, bleeding, and dysuria. Cervical cancer begins at the bottom of the cervical tissue and progresses as it spreads to other places [11]. In the second stage, the abnormal cells spread to the vagina, although the recovery rate remains high. In the third stage, the abnormal cells spread to the lymph nodes in the pelvis and vagina and cause kidney problems. At this stage, the chance of recovery becomes low. In the fourth stage, abnormal cells spread outside the pelvis and invade the bladder and other nearby organs. This stage is difficult to treat. At this stage, the patient only receives pain-relieving health care [12]. There are several techniques for cervical cancer screening that have led to a lower mortality rate. There are consistent techniques for detecting abnormal cervical intraepithelial cells, such as smear techniques. There are also techniques for examining the cervix, such as liquid-based cytology (LBC), microscopy, digital colposcopy, and HPV testing [13]. The LBC is a method of collecting and preparing cervical cells for testing. It is a newer method than the traditional Pap smear, and it has several advantages. In a traditional Pap smear, a sample of cells is taken from the cervix and smeared onto a glass slide. The slide is then stained and examined under a microscope by a cytotechnologist or pathologist. In LBC, the cells are collected in a liquid preservative solution. The solution is then centrifuged, which separates the cells from the liquid. The cells are then spread onto a slide and stained. LBC has several advantages over traditional Pap smears. It is more sensitive, meaning that it is more likely to detect abnormal cells. It is also more specific, meaning that it is less likely to produce false-positive results. LBC is also less likely to be affected by blood or other debris, which can make it difficult to interpret traditional Pap smears. LBC is the preferred method of cervical cancer screening in many countries. It is more effective than traditional Pap smears, and it is less likely to be affected by blood or other debris. Thus, LBC technology played a key role in analyzing cervical smears and detecting the type of cervical cancer to help patients receive appropriate health care. The doctor magnifies the LBC slice under the microscope to different magnification factors such as 400×. Therefore, the doctor obtains thousands of cells, which must be carefully examined [14]. In the initial stages, a few abnormal cells may appear that doctors may not focus on, causing a wrong diagnosis. The gap between the number of experienced doctors and patients is also one of the challenges facing health care. The examination of LBC slides is codified and time-consuming, and it is subject to differing doctors’ opinions about the diagnosis. Thus, automated computer-assisted techniques solve the challenges of manual diagnosis. The WSIs are a method of numbering glass slides obtained with WSI scanners [15]. WSI allows for the digital viewing and analysis of tissue sections. Thus, the advent of WSI images led to the use of machine and deep learning (DL) to analyze images of cervical cells to assist doctors in analyzing WSI images, segmenting, and classifying cells, as well as predicting the type of cervical cancer.

Early diagnosis plays a crucial role in the effective management and treatment of cervical cancer. The WSI has emerged as a powerful tool in the field of pathology, allowing for the digitization and analysis of histopathological slides. However, the quality of WSI images can significantly impact the accuracy and reliability of diagnostic assessments. One fundamental aspect of improving WSI image quality is enhancing resolution. Higher-resolution images provide finer details and enable pathologists to better visualize cellular structures and tissue morphology. Various techniques can be employed for resolution enhancement. These techniques aim to reconstruct high-resolution images from low-resolution counterparts, thus enhancing WSI images’ overall quality and diagnostic potential. Noise, such as random variations in pixel values, can degrade the quality of WSI images and introduce artifacts that may hinder accurate diagnosis. Effective noise reduction techniques, such as spatial filtering and wavelet denoising, can be applied to minimize noise while preserving important image features. By reducing noise, the clarity and visibility of cellular structures and pathological characteristics are improved, aiding pathologists in making accurate assessments. WSI images may contain artifacts, including dust particles, stains, and tissue folds, which can obstruct the visibility of critical features. These artifacts can be removed using various techniques, such as morphological operations, adaptive filtering, and inpainting algorithms. By effectively eliminating artifacts, the clarity and accuracy of WSI images are enhanced, enabling pathologists to focus on relevant structures and abnormalities. Enhancing the contrast of WSI images can improve the visibility of subtle features and facilitate the identification of abnormal tissue patterns. Contrast enhancement techniques, such as histogram equalization, adaptive contrast stretching, and retinex-based methods, can be employed to amplify the differences in pixel intensity values across the image. This enhancement enables better discrimination between healthy and abnormal tissue regions, aiding in the early detection and diagnosis of cervical cancer. Improving the quality of WSI images is vital for early diagnosis and the accurate assessment of cervical cancer. By employing techniques such as resolution enhancement, noise reduction, color correction, artifact removal, contrast enhancement, and image registration, the overall quality and diagnostic utility of WSI images can be significantly improved. These candidates provide valuable avenues for research and development, aiming to advance the field of early detection and diagnosis of cervical cancer using WSI technology. DL networks play a vital role in analyzing WSI images to extract salient and hidden characteristics, as well as biomarkers, that cannot be detected by manual diagnosis. There is a similarity between the characteristics of the types of cervical cancer, especially in the early stages. Therefore, to receive the appropriate treatment, DL networks have the superior ability to extract the characteristics of each type of cervical cancer to help doctors distinguish between types of cervical cancer [16]. In this study, to solve the challenge of the similarity of cervical cancer types, the focus was on extracting features from more than one CNN model, integrating them sequentially into vectors and classifying them using RF and SVM networks.

Here, previous studies on techniques were used to analyze the images of the cervical cancer dataset and the results that were reached.

Kavitha et al. [17] applied a technique to improve cervical images by Dynamic Fuzzy Histogram Equalization and to identify regions of interest by fuzzy c-mean. The ant colony optimization method was applied to select and classify the traits by CNN and ANN. Badiea et al. [18] applied a CNN hybrid method with SVM for the analysis of WSI images of the cervix. CNN layers extracted the features, and PCA selected the features. The SVM based on the GoogLeNet features achieved an accuracy of 96.8%. Hiam et al. [19] used a method for integrating DL features with Shuffle Net to classify cervical cancer. PCA and canonical correlation (CCA) methods were applied to obtain discriminatory features for each class. The discriminatory features of the CCA method were classified by ML methods SVM, RF, and ANN, which reached an accuracy of 91.1%, 94.7%, and 94.9%, respectively. Jesse et al. [20] analyzed cervical cancer risk factors using the decision tree algorithm. A recursive feature elimination (RFE) method was applied to select the predictive characteristics of cervical cancer. SMOTE Tomek was applied to collate the dataset. The decision tree reached an accuracy of 95.29%. Odai et al. [21] presented a system for predicting cervical cancer using the Genetic Method (GM), PCA, and MLP Algorithm. The GM optimizes the parameters of the MLP, and the prediction is simulated within the GM by the MLP. The features were fed into several classifiers, where the RF achieved an accuracy of 92.16%, a specificity of 85.57%, and a precision of 87.22%. Mingmei et al. [22] applied an unsupervised ML method for analyzing cervical cytology images. The images improved, and the PCA was applied to represent the features in a low-dimensional space. The method yielded a sensitivity of 81.8% and a specificity of 86.5%. Park et al. [23] applied a DL and ML method for cervical cancer detection. The images were optimized, and 300 features were extracted and classified by XGB and SVM. Validated tests and ROC analysis yielded an AUC of 82% and 84% for both XGB and SVM. Yoon et al. [24] used a CNN based on acetowhite for cervical cancer detection. A comparison was made between the method before and after improvement in the area of interest, where the method based on acetowhite reached an accuracy of 81.31%. Naif et al. [25] developed several ML methods based on the previous stages: image optimization and selection of vital features for each class by predictive model selection (PMS). Phasit et al. [26] developed an RF-based cervical cancer prediction model called iPMI. Features were extracted along with clinical characteristics with characteristics of cervical neighboring regions called iPMI-Econ. The iPMI–Power method showed a superiority of 86.2%, a sensitivity of 60%, and an AUC of 90.5%. Madhura et al. [27] applied a hybrid method between machine learning and fuzzy min–max neural to analyze and extract features from cervical images. The method took advantage of fuzzy min–max neural features, as Resnet-50 reached an accuracy of 95.33%. Deborah et al. [28] featured an analysis of the nuclei of cervical cells and their classification by a hierarchical method of ML algorithms. Quality results were obtained through a hierarchical classification with RF, which yielded an accuracy of 95.34%, a precision of 84.31%, and a sensitivity of 84%. Mohammed et al. [29] applied a DL network for the analysis of all-cell of WSI images of the cervix. A median filter has been applied to improve the images and augment the data to balance the classes. The systems performed better after the image improvement process. Débora et al. [30] used DL models for analyzing cervical cells for cervical cancer detection. Deep features were extracted from the cervical cancer dataset and classified, where the XceptionNet model yielded an accuracy of 93%, sensitivity of 80%, and specificity of 80%. Shervan et al. at [31] applied three deep networks to extract features and replace the classification layers with MLP, KNN, and RF networks. The MLP, KNN, and RF networks resulted in an accuracy of 96.53%, 94.43%, and 95.51%, respectively.

Nitin et al. [32] proposed a hybrid deep feature concatenated network (HDFCN) for the detection of cervical cancer in WSI images. The network combined features from three deep-learning models. WSI images were enhanced to high resolution and resized to pixels to reduce computational costs and fit into deep-learning models. The performance of the proposed HDFCN model was compared to individual deep-learning models. The results showed that the HDFCN model outperformed the individual models in classifications, achieving an accuracy of 97.45%. Ishak et al.’s [33] study employed state-of-the-art deep-learning techniques, specifically in two categories: CNN approaches and vision transformer (ViT) approaches. To make the models efficient, WSI images were optimized to high resolution and resized to a manageable number of pixels. The experimental results showed that the latest ViT-based models outperformed the CNN models, while the existing CNN models performed similarly to the ViT models. By leveraging data augmentation and ensemble-learning techniques in ViT-based models, the research achieved a level of success. Yuan et al. [34] aimed to address the grade classification problem on segmented epithelium patches using conditional Generative Adversarial Networks (cGANs). The study introduced a synthetic-image filtering mechanism based on the divergence in feature space between the generated images and class centroids. This filtering process aimed to control the quality of selected synthetic images for data augmentation, ensuring that only meaningful features were included. The results demonstrated a significant improvement in classification accuracy. Specifically, the classification accuracy increased from 66.3% to 71.7% when utilizing the cGAN generated images with feature-based filtering.

It is noted that researchers devoted their efforts to designing techniques with various tools to reach satisfactory results. However, because of the similarity of cervical cancer cell types in their early stages, diagnostic accuracy is still the goal of every researcher. This study focuses on various techniques for extracting fused features by CNN models and replacing the classification layers of CNN with other algorithms. This study reached satisfactory results compared to previous related studies, which is due to the extraction of fused features between CNN models and the application of dimension reduction techniques to select important features only.

The main contributions of this study are as follows:

- Applying two successive filters to improve the quality of WSI images and show the edges of low-contrast cells.
- Applying the ACA algorithm to separate the cells to be analyzed, separating them from the rest of the image, and saving them in a new file to feed them into DL models.
- Developing a hybrid technique between machine-learning algorithms (RF and SVM) and ResNet50, VGG19, and GoogLeNet models for analyzing WSI images for cervical cancer diagnosis.
- Developing a technique to extract the fused features of DL models (ResNet50-VGG19, VGG19-GoogLeNet and ResNet50-GoogLeNet) and classify them by RF and SVM algorithms.

The remainder of the study is arranged as follows: Section 2 summarizes the methods and results of several previous studies. Section 3 explains the various techniques and tools applied to analyze WSI images of abnormal cells of cervical cancer. Section 4 presents the results of the proposed approaches in the study. Section 5 compares the performance of the proposed approaches and concludes with the best performance compared to the studies mentioned in Section 2. Section 5 concludes the study.

## 2. Materials and Methods

### 2.1. Description of the Cervical Squamous Cell Dataset

In this study, the performance of the developed systems was measured using the cervical intra-epithelial squamous cell (CESC) dataset. LBC is an intrinsic technique for cervical cancer screening. Glass slides were converted into WSI images for computer-aided analysis. The dataset consists of 962 images acquired with a Leica ICC50 HD microscope at a magnification factor of 40×. The dataset is distributed among four classes representing the stages of squamous cell development of cervical cancer as follows: 163 images of a high-squamous intra-epithelial lesion (HSEL), 113 images of a low-squamous intra-epithelial lesion (LSEL), 612 images of negative for intra-epithelial malignancy (NEM), and 74 images of a squamous cell carcinoma (SCC) [35]. Doctors and specialists examine abnormal cell changes to detect malignant or pre-malignant features. Therefore, it takes a long time, and the diagnosis is subject to different opinions. Therefore, computer-assisted techniques limit the late diagnosis of cervical cancer and help doctors support their diagnosis to administer appropriate treatment before it is too late.

### 2.2. Enhancement of WSI Images of the CESC Dataset

The introduction chapter should discuss filters to improve WSI image quality.

Image enhancement is one of the most important processes in image processing to improve certain features to make the image clearer. In the optimization process, artifacts, which the system considers as features if not deleted, are removed. Optimization methods provide a mechanism for interpreting information for experts and are the first step in medical image processing [36]. The main goal of optimizing medical images is to modify some features and make them visible for processing in the next stages. In image processing, the edges of regions of interest (ROI) are highlighted, and the low contrast between ROI and other regions is processed. In this study, a Gaussian filter was applied to remove the artifacts and a Laplacian filter to show the edges of the ROI.

First, the images were fed to an averaging filter with a 6 × 6 operator factor. Each time, one image is taken, and each pixel is processed based on 35 adjacent pixels. The average filter works each time the target pixel is selected, and the average values of 35 adjacent pixels is calculated [37]. The average value of the neighbors then replaces the target pixel value. The process continues until every image pixel has been processed, as in Equation (1).

With input image *h*(*x*, *y*) and the filter size of *M* = *m* × *n* = 6 × 6, the output of an averaging filter at *O*(*x*, *y*) is

(1) $$O(x,y)=\frac{1}{M}\sum_{i=-c}^{c}\sum_{j=-d}^{d}h(x+i,\;y+j)$$

where *x* and *y* are changed so that the target of the filter wraps every pixel in *f* once, and *m* = 2*c* + 1, *n* = 2*d* + 1.

After removing the artifacts, there remains a low contrast. Some images show a low contrast between the infected and healthy areas. Therefore, the images were passed through a Laplacian filter to detect the edges [38]. The Laplacian filter uses the second derivative to show the areas of change and increase the contrast of the edges, as in Equation (2).

(2) $$\nabla^{2}\;f=\frac{\partial^{2}\;f}{\partial^{\;}x^{2}}+\frac{\partial^{2}\;f}{\partial^{\;}y^{2}}$$

where: *x* and *y* as equation in (3) and (4)

(3) $$\frac{\partial^{2}\;f}{\partial^{\;}x^{2}}=f\left(x+1,y\right)+f\left(x-1,y\right)-2f\left(x,y\right)\;$$

(4) $$\frac{\partial^{2}\;f}{\partial^{\;}y^{2}}=f\left(x,y+1\right)+f\left(x,\;y-1\right)-2f\left(x,y\right)$$

Finally, the outputs of the average and Laplacian filters are integrated to obtain an improved image, as in Equation (5).

(5) $$e(x,\;y)=O(x,y)-\nabla^{2}\;f$$

where e(x, y) is the enhanced WSI image.

**Figure 1:**
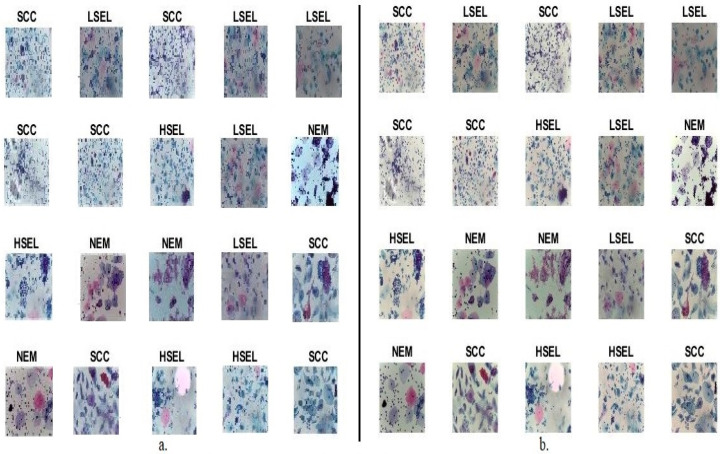
Random images from a CESC dataset for cervical cancer (**a**) Before improvement (**b**) After improvement.

Figure 1 shows random samples of CESC dataset images of all stages of cervical cancer development before and after enhancement.

### 2.3. Active Contour Algorithm

The segmentation stage is an essential process in medical image processing, and it is a difficult process that must be fast and accurate. The segmentation process is difficult because of the challenges faced by the systems, such as low contrast, reflections, and irregular borders [39]. CNN models extract features from whole images. In this study, the regions required for analysis in the following stages were extracted by the ACA method in order to feed the CNN models with ROI regions only. The WSI images contain two parts: the affected cells to be analyzed, called ROIs, and the healthy parts between cells. If the segmentation is done correctly, the CNN models will extract the most efficient and effective features to represent each type of cervical cancer stage.

The active contour algorithm segments the WSI images to calculate the ROI for further analysis. An active perimeter is a model that separates desired areas from the background to obtain efficient results. ACA provides a smooth and close environment for ROI boundary extraction. A contour is a boundary defining an area required for analysis. It is a set of points that are subject to the interpolation process, which is polynomial or linear and has the ability to specify the required areas. Active contour is a method for obtaining deformable structures, which describe the required bounds of curve formation and contour parameters. Internal and external forces are applied to determine the curve models as in Equation (6). These forces are related to the curves of the image. External energy controls the boundaries of the image through a mixture of energy in the vicinity of areas within the image, while internal energy controls deformable changes [40].

(6) $$G_{cv}C=\int_{outside}(I(x)-m_{1}{)}^{2}\;dx+\int_{inside}\left(I(x)-m_{2}{)}^{2}\;dx+\beta length\;(C\right)$$

where *I* refers to gray intensity, *outside* and *inside* refer to the areas inside and outside the contour *C*, *m*_1_ and *m*_2_ refer to intenseness outside and inside, *β* refers to a parameter, and *length*(*C*) refers to the length of contour *C*. *G_CV_* has less initialization sensitivity and few parameters to adjust. *G* makes the contour develop to the edge of the object [41].

**Figure 2:**
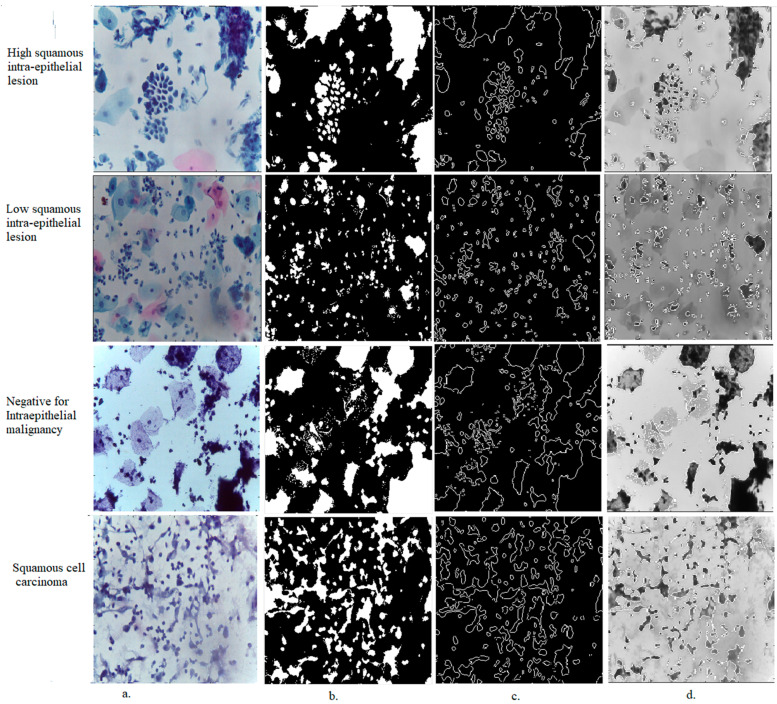
Samples from all classes of the CESC dataset for cervical cancer after segmentation by the ACA method (**a**) Original images (**b**) Segmentation (**c**,**d**) Region of interest.

ACA is used in medical image segmentation to extract the desired pixels for analysis at high resolution. Algorithm 1 describes how ACA segments WSI images to extract the desired cells to be analyzed. Figure 2 shows a sample set of CESC dataset images for all classes after applying ACA. Thus, all CESC dataset images were segmented and fed into CNN models to extract features from the ROI regions.
**Algorithm 1:** Segmentation of WSI images of CESC Dataset by ACA methodStep 1:Input: Enhanced ImageStep 2:Convert the Image to Gray doubleStep 3:Initial contour C0Step 4:For L = 0 to the maximum number of iterations doStep 5:$G_{cv}C=\int_{outside}(I(x)-m_{1}{)}^{2}\;dx+\int_{inside}\left(I(x)-m_{2}{)}^{2}\;dx+\beta length\;(C\right)$Step 6:Discover the regions inside and outside the contour 𝐶 Step 7:Extract energy through image informationStep 8:Gradient descent to minimize energyStep 9:Evolve the curveStep 10:End forStep 11:Output: WSI images of regions of interest only

### 2.4. Training of Hybrid Systems with Combined CNN Features

#### 2.4.1. CNN for Deep-Feature Extraction

Convolutional neural networks (CNNs) are used in various fields, including healthcare and biomedical image analysis. CNN is distinguished from traditional neural networks in that each neuron is transformed into a 2D or 3D filter based on the input layer [42]. CNN offers different layers, such as convolutional layers, which are the most important and relevant. Convolutional layers are a set of filters that wrap around an input, resulting in feature maps being produced as output as in Equation (7). CNN uses the image and applies filters to it while extracting deep features that represent the image [43]. After the convolutional layers, the ReLu function is used, which converts negative values to zero. There are two ways to adjust the image size in convolutional layers. First, padding, which is adding zeros to the edges of the image to preserve the size of the original image [44]. For example, when passing an 8 × 8 image over a 4 × 4 filter, the output is 8 × 8. Thus, the input dimension is preserved. Secondly, Stride is a parameter that controls the jump of the filter on the image at each iteration. The higher the Stride value, the less information is analyzed. In addition, the computation time is reduced [45]. If the value of Stride is low, more information will be analyzed to obtain large outputs, and the processing time needs to be increased [38].

(7) $$y\left(t\right)=\left(x*f\right)\left(t\right)=\int x\left(a\right)f\left(t-a\right)\;\;da\;$$

where *f*(*t*) is the filter, *x*(*t*) and *y*(*t*) indicate the input and output image.

Convolutional layers produce a high-dimensional image size, requiring complex and time-consuming calculations. Therefore, CNN provides pooling layers that reduce the image size, thus reducing the computational cost. Pooling layers aim to maintain feature maps with the most important features [37]. There are two ways to reduce the size of an image:

Max-pooling: the image is divided into many regions, and each region contains a group of pixels (neuron values). The pixel with the max value is extracted from each region as in Equation (8) [46].

Average-pooling: the image is divided into many regions, each containing a set of pixels. The average value of each region is calculated, and all pixels of the region are replaced by their average value, as in Equation (9) [47].

(8) $$z\left(i;\;j\right)={max}_{m,n=1\ldots k}\;f\left[\left(i-1\right)p+m;\;\left(\;j-1\right)p+n\right]\;$$

(9) $$z\left(i;\;j\right)=\frac{1}{k^{2}}\sum_{m,n=1\ldots k}f\left[\left(i-1\right)p+m;\;\left(\;j-1\right)p+n\right]\;$$

where *f* is filter size, *p* is Filter wrap, *m*, *n* is the matrix location, and *k* is the vectors.

Thus, the main task of ResNet50, VGG19, and GoogLeNet models is to extract and save features in feature vectors. The classification layers were removed from the models, and the convolutional, pooling, and auxiliary layers were preserved. ResNet50, VGG19, and GoogLeNet models generated high-level feature maps as follows: (3, 3, 512), (7, 7, 512), and (7, 7, 512), respectively. The high-level feature maps were converted into single-feature vectors for each image by the global average pooling (GAP) layer and saved in feature vectors of size 2048, 4096, and 4096 for ResNet50, VGG19, and GoogLeNet, respectively. Thus, the CESC dataset features are 1296 × 2048, 1296 × 4096, and 1296 × 4096 for ResNet50, VGG19, and GoogLeNet, respectively.

It is noted that the extracted features are high dimensional. Therefore, the PCA algorithm was applied. PCA reduces dimensionality by selecting important features, deleting redundant features and strongly correlated features to keep one correlated feature. Thus, the size of the CESC dataset becomes 1296 × 615, 1296 × 720, and 1296 × 795 for ResNet50, VGG19, and GoogLeNet, respectively.

#### 2.4.2. Classification

The classification stage accurately interprets the analyzed data through the previous image processing stages. In the classification phase, the dataset is divided into training and testing [48]. In the training stage, the network is trained on features extracted from the previous stage and saved in feature vectors, with a label for each class. The testing stage is the most important stage for knowing the classifier’s performance, and the network is evaluated by testing it on a set of new features. CNN models require high specifications, expensive computers, and long implementation times. In addition, transferring the learning of the CNN models to training the CESC dataset is not satisfactory because the pre-trained CNN models were trained on the ImageNet dataset that does not contain medical images [49]. Therefore, a hybrid technique of CNN-RF and CNN-SVM was applied to solve these challenges. The methodology consists of two parts: CNN models (ResNet50, VGG19, and GoogLeNet) to extract features with high accuracy; RF and SVM networks to classify features of CNN models [50].

##### Random Forest Algorithm

Random Forest is a machine-learning algorithm that combines several decision tree results. RF is easy to use, flexible, and handles many classification and regression tasks. Thus, many decision trees form the RF, which becomes more accurate. The RF algorithm is based on the principle of ensemble learning, which is based on classification by many decision trees. Then it aggregates all the predictions of the decision trees to get one more accurate result. The most ensemble method is bagging, which is called boosting. In the bagging method, the training data is randomly selected with replacement. This means selecting training samples more than once and training the models independently to perform the classification task. RF is an extension of the bagging method, which uses the bagging method and the randomness feature to generate the RF [51]. Feature randomness is also known as feature bagging, where a set of features is randomly generated to ensure little correlation between decision trees. Three main parameters need to be set before training: the node size of each tree, the number of decision trees, and the number of features. In this study, samples have been selected from the CESC dataset to classify the dataset into four classes (stages of cervical cancer development) [52].

The RF is fed with the features of ResNet50, VGG19, and GoogLeNet models in sizes 1296 × 615, 1296 × 720, and 1296 × 795 separately. RF divides the data into training data and then evaluates RF performance through test data.

##### SVM Algorithm

SVM is a supervised machine-learning algorithm used to solve classification and regression problems. The algorithm’s goal is to create a line or boundary to separate the features of an n-dimensional dataset into classes to classify new data points into the appropriate category easily. The decision boundary separating the classes is called a hyperplane. The SVM helps create the hyperplane through extreme data points called support vectors. Two types of SVM are linear and nonlinear. The linear SVM type is used when the data are linearly separable by a single hyperplane. Nonlinear SVM is used when data are not linearly separable [53]. SVM creates several decision boundaries for separating n-dimensional dataset classes and chooses the decision boundary with a max margin between the closest data points of different classes, called the SVM hyperplane [54].

The SVM is fed with the features of ResNet50, VGG19, and GoogLeNet models in sizes 1296 × 615, 1296 × 720, and 1296 × 795 separately. SVM divides the data into training data and then evaluates SVM performance through test data.

**Figure 3:**
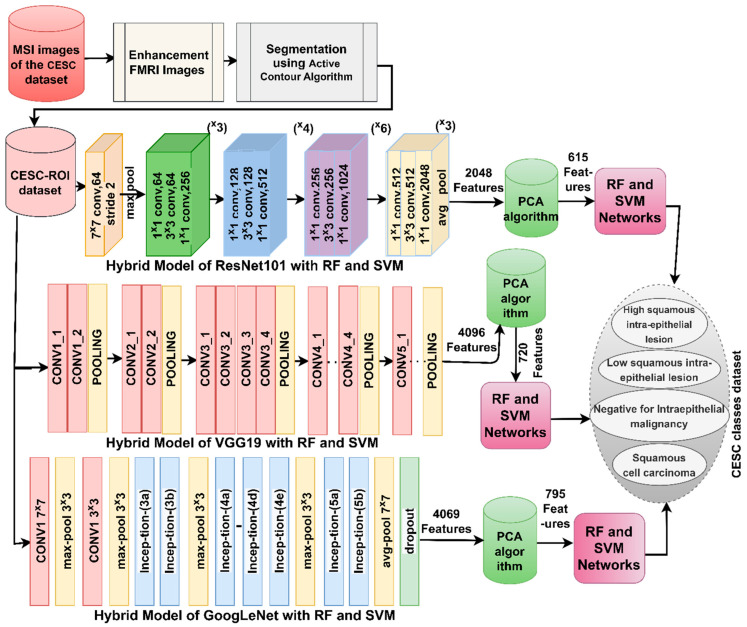
Strategies of WSI image analysis for the CESC dataset for cervical cancer by CNN-RF and CNN-SVM.

In this hybrid methodology, the first model, shown in Figure 3, was implemented through the following sequence: first, WSI images of the CESC dataset were optimized. Second, the ACA method was applied to separate the cells to be analyzed, isolate them from the rest of the image, and save them as the CESC–ROI dataset. Third, ResNet50, VGG19, and GoogLeNet models analyze required cells to extract representative feature maps for each image and save them in sizes 1296 × 2048, 1296 × 4096, and 1296 × 4096 for ResNet50, VGG19, and GoogLeNet, respectively. Fourth, feature maps are moved to PCA to reduce dimensionality by selecting important features, deleting highly correlated features, keeping only one, and saving them in sizes 1296 × 615, 1296 × 720, and 1296 × 795 for ResNet50, VGG19, and GoogLeNet, respectively. Fifth, important features are fed into the RF and SVM networks, which work to divide them during the training phase and keep feature vectors randomly during the testing phase to test the performance of the systems.

**Figure 4:**
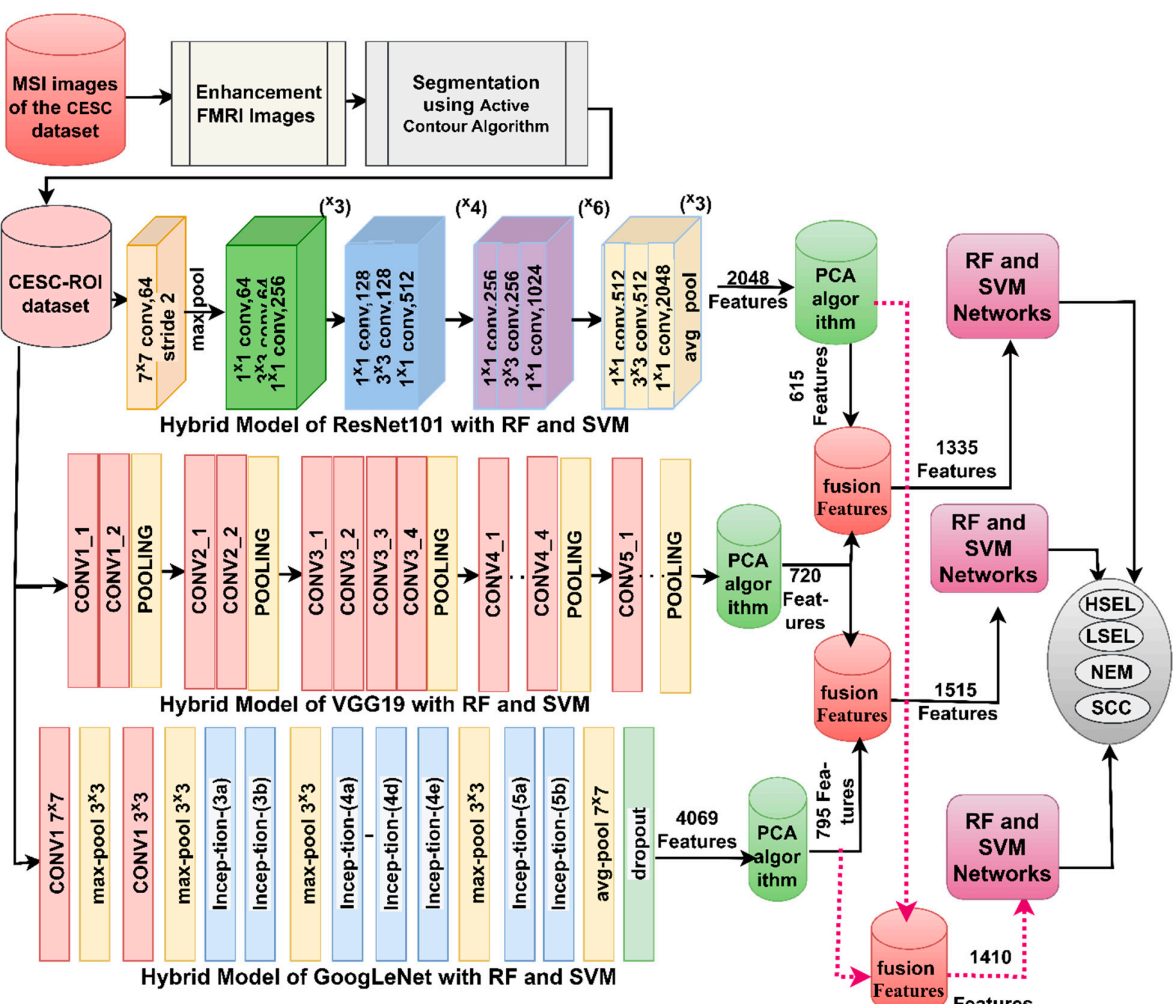
Strategies of WSI image analysis for CESC dataset for cervical cancer by RF and SVM with integrated CNN features.

The second model, shown in Figure 4, is a hybrid technology based on combining features of CNN models and their classification by RF and SVM networks, which were implemented through the following sequence. The four sequential stages are the same as the first model. The fifth stage integrates the features produced by ResNet50, VGG19, and GoogLeNet models in a series as follows: ResNet50-VGG19, VGG19-GoogLeNet, and ResNet50-GoogLeNet. They are then saved in feature vectors with sizes 1296 × 1335, 1296 × 1515, and 1296 × 1410. Sixth, the hybrid CNN feature vectors are placed in the RF and SVM networks for highly accurate classification.

## 3. Results of System Performance

### 3.1. Separating CESC Dataset

In this study, the CESC dataset was used to measure the quality of the proposed systems. The dataset contains 962 images at 40× magnification distributed over four unbalanced classes, as shown in Table 1. All systems randomly divided CESC dataset images into 80% for training and validation (80:20) and isolated 20% of the CESC dataset for system testing.

### 3.2. Performance Measures

The performance of the systems was evaluated using the metrics mentioned in Equations (10)–(14). The evaluation equations contain variables that refer to correctly classified images called TP and TN and images that belong to a class but are classified as another class called FN and FP. Systems produce a confusion matrix, which is the most important criterion for measuring the performance of systems. The correctly and incorrectly classified images indicated in the equations are extracted through the confusion matrix.

(10) $$AUC=\frac{TP\;Rate}{FP\;Rate}$$

(11) $$Sensitivity=\frac{TP}{TP+FN}*100\%$$

(12) $$Accuracy=\frac{TN+TP}{TN+TP+FN+FP}*100\%$$

(13) $$Precision=\frac{TP}{TP+FP}*100\%$$

(14) $$Specificity=\frac{TN}{TN+FP}*100$$

### 3.3. Handling Lacks and Balancing of Data

**Figure 5:**
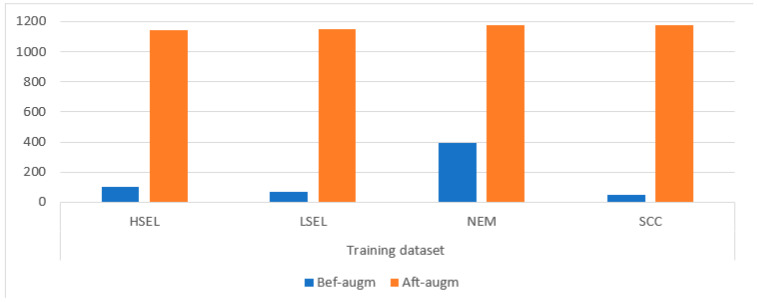
Dataset balancing by augmenting images of the dataset by the data-augmentation method.

One of the major limitations of CNN models is the lack of medical images when training models. In order to achieve good results, CNN models need a lot of images, and unfortunately, there are not enough medical images available to train the CNN models [55]. The second limitation of CNN models is the imbalanced dataset, which contains different images for each class. Therefore, these limitations must be overcome. The data-augmentation method was applied to handle the two limitations. Data augmentation artificially produces images from the same dataset through many operations, such as horizontal and vertical flipping, rotating at multiple angles, displacement, resizing, cropping, etc. Thus, the first limitation was processed [56]. To address the second constraint, the increase in the number of images must be different between the classes. Therefore, the images of the majority class are increased by a smaller amount than the minority class. Thus, the second limitation was overcome, and the dataset has been balanced. Table 2 and Figure 5 summarize the distribution of the number of images of the CESC dataset before and after data augmentation was applied. It is noted from the table that the WSI images of the HSEL class were increased by 10 times for each original image. The WSI images of the LSEL class were increased by 16 times for each original image. The WSI images of the NEM class were increased thrice for each original image. The WSI images of the SCC class were increased by 25 times for each original image.

### 3.4. Results of CNN Models

This section summarizes the results of the pre-trained CNN models. It is worth noting that these models were trained on more than a million images in the ImageNet dataset. Still, despite the huge number of images, some biomedical images were unavailable in the ImageNet dataset. Thus, the transfer learning of CNN models through the ImageNet dataset was transferred to perform new tasks on the cervical cancer dataset. The improved images are fed to ResNet50, VGG19, and GoogLeNet models for analysis, feature extraction through convolutional layers, dimensionality reduction by pooling layers, and classification by fully connected layers.

**Figure 6:**
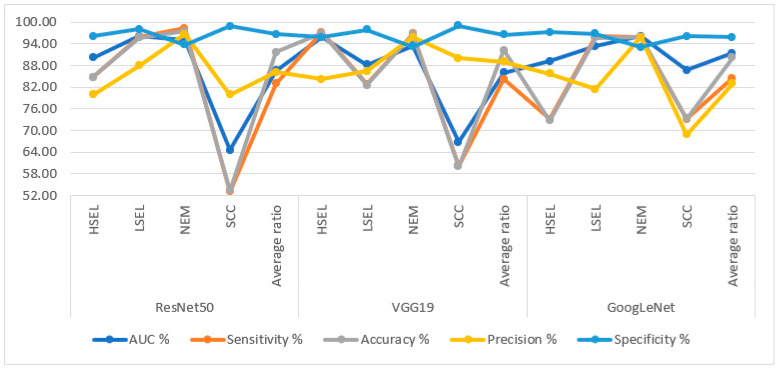
Display of performance results of DL models for analysis, and WSI image of a CESC dataset for the diagnosis and discrimination of cervical cancer.

Table 3 and Figure 6 summarize the performance of the ResNet50, VGG19, and GoogLeNet models for analyzing WSI images for diagnosing and differentiating stages of the cervical cancer dataset. ResNet50 has reached an AUC of 86.53%, a sensitivity of 83.03%, an accuracy of 91.70%, a precision of 86.18%, and a specificity of 96.75%. Meanwhile, the performance of the VGG19 has reached an AUC of 86.10%, a sensitivity of 84.30%, an accuracy of 92.20%, a precision of 89.13%, and a specificity of 96.53%. In contrast, GoogLeNet achieved an AUC of 91.38%, a sensitivity of 84.58%, an accuracy of 90.20%, a precision of 82.98%, and a specificity of 95.83%.

### 3.5. Results of CNN Based on the ACA Algorithm

The section summarizes the performance results of the pre-trained ResNet50, VGG19, and GoogLeNet models based on the ACA hashing algorithm. The WSI images of the abnormal cells of the cervix were enhanced and then fed to the ACA algorithm to segment the cells to be analyzed and separate them from the other unwanted areas. Thus, ResNet50, VGG19, and GoogLeNet models were fed with important cells to be analyzed. The segmented images are analyzed by convolutional layers, and their dimensions are reduced by pooling layers. Finally, the high-level features are converted to single-level features by fully connected layers, and each image is labelled to its appropriate class through the SoftMax function.

**Figure 7:**
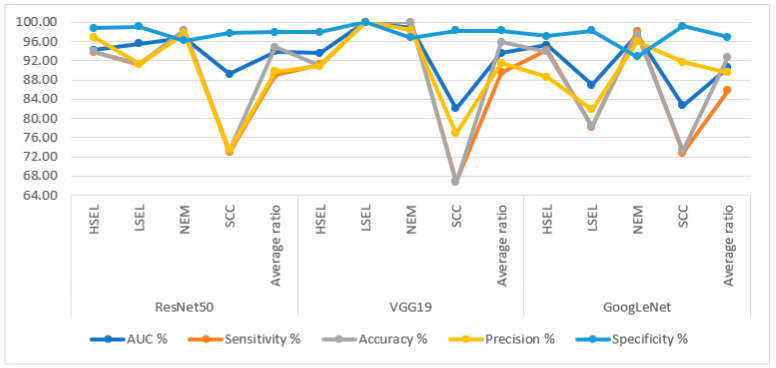
Display of performance results of DL models based on ACA algorithm for analysis, and WSI image of a CESC dataset for the diagnosis and discrimination of cervical cancer.

Table 4 and Figure 7 summarize the performance of ResNet50, VGG19, and GoogLeNet models for WSI image analysis based on the ACA algorithm for diagnosing and differentiating stages of cervical cancer dataset. ResNet50 has reached an AUC of 93.9%, a sensitivity of 89%, an accuracy of 94.8%, a precision of 89.78%, and a specificity of 97.95%. Meanwhile, the performance of the VGG19 has reached an AUC of 93.63%, a sensitivity of 89.53%, an accuracy of 95.9%, a precision of 91.55%, and a specificity of 98.23%. In contrast, GoogLeNet achieved an AUC of 90.55%, a sensitivity of 85.83%, an accuracy of 92.7%, a precision of 89.53%, and a specificity of 96.85%.

### 3.6. Results of Hybrid Systems

The section summarizes the performance of hybrid methodologies for DL models (ResNet50, VGG19, and GoogLeNet) with RF and SVM algorithms based on the ACA algorithm. The methodology consists of two blocks. The first block includes DL models that conduct feature extraction and then reduction via PCA. The second block is RF and SVM algorithms, which receive representative features, separate them into training and validation data, and isolate 20% for systems testing. The hybrid DL-RF and DL-SVM methods have superior capabilities for analyzing WSI images of cervical cancer and discriminating between stages of their progression.

**Figure 8:**
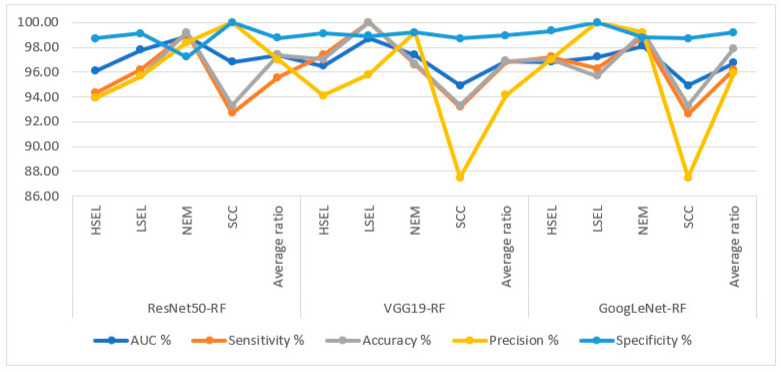
Display of performance results of DL with RF based on ACA algorithm for analysis, and WSI image of a CESC dataset for the diagnosis and discrimination of cervical cancer.

Table 5 and Figure 8 summarize the performance of hybrid methodologies for DL models with the RF algorithms. WSI images are analyzed based on the ACA algorithm for diagnosing and differentiating stages of cervical cancer development. ResNet50 with RF reached an AUC of 97.4%, a sensitivity of 95.58%, an accuracy of 97.4%, a precision of 97%, and a specificity of 98.75%. The performance of the VGG19 with RF reached an AUC of 96.88%, a sensitivity of 96.8%, an accuracy of 96.9%, a precision of 94.14%, and a specificity of 98.98%. In contrast, GoogLeNet with RF reached an AUC of 96.75%, a sensitivity of 96.23%, an accuracy of 97.9%, a precision of 95.93%, and a specificity of 99.2%.

**Figure 9:**
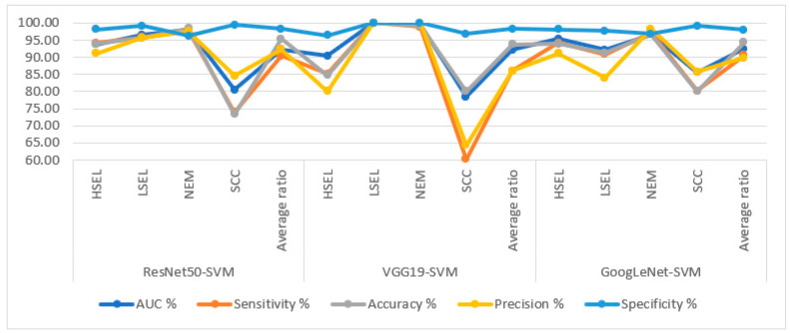
Display of performance results of DL with SVM based on ACA algorithm for analysis, and WSI image of a CESC dataset for the diagnosis and discrimination of cervical cancer.

Table 6 and Figure 9 summarize the performance of hybrid methodologies for DL models with the SVM algorithms. ResNet50 with SVM reached an AUC of 92.28%, a sensitivity of 90.43%, an accuracy of 95.3%, a precision of 92.28%, and a specificity of 98.25%. The performance of the VGG19 with SVM reached an AUC of 92.1%, a sensitivity of 86.08%, an accuracy of 93.8%, a precision of 86.1%, and a specificity of 98.3%. In contrast, GoogLeNet with SVM reached an AUC of 92.45%, a sensitivity of 90.6%, an accuracy of 94.3%, a precision of 89.8%, and a specificity of 97.95%.

**Figure 10:**
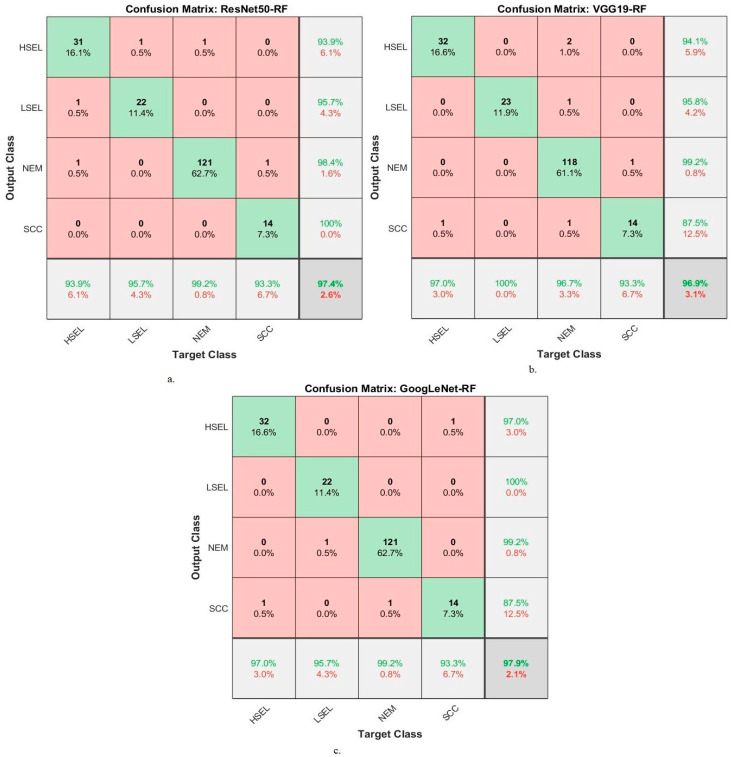
Confusion matrix for display performance results of DL with RF based on ACA algorithm for analysis, and WSI image of a CESC dataset for the diagnosis and discrimination of cervical cancer.

The hybrid methodologies of DL-RF produce confusion matrices that measure the performance of the hybrid methods for early diagnosis of cervical cancer and differentiation between stages of its development. Figure 10 displays the confusion matrix for the ResNet50-RF, VGG19-RF, and GoogLeNet-RF models for analyzing WSI images for cervical cancer detection. The figure shows the accuracy reached by the ResNet50-RF system for each class: 93.9% for HSEL, 96.7% for LSEL, 99.2% for NEM, and 93.3% for SCC. While for the VGG19-RF system, the accuracy for each class was achieved as follows: for HSEL class of 97%, for LSEL class of 100%, for the NEM class of 96.7%, and for the SCC class of 93.3%. Meanwhile, the GoogLeNet-RF system resulted in accuracy for each class as follows: 97% for HSEL, 95.7% for LSEL, 99.2% for NEM, and 93.3% for SCC.

**Figure 11:**
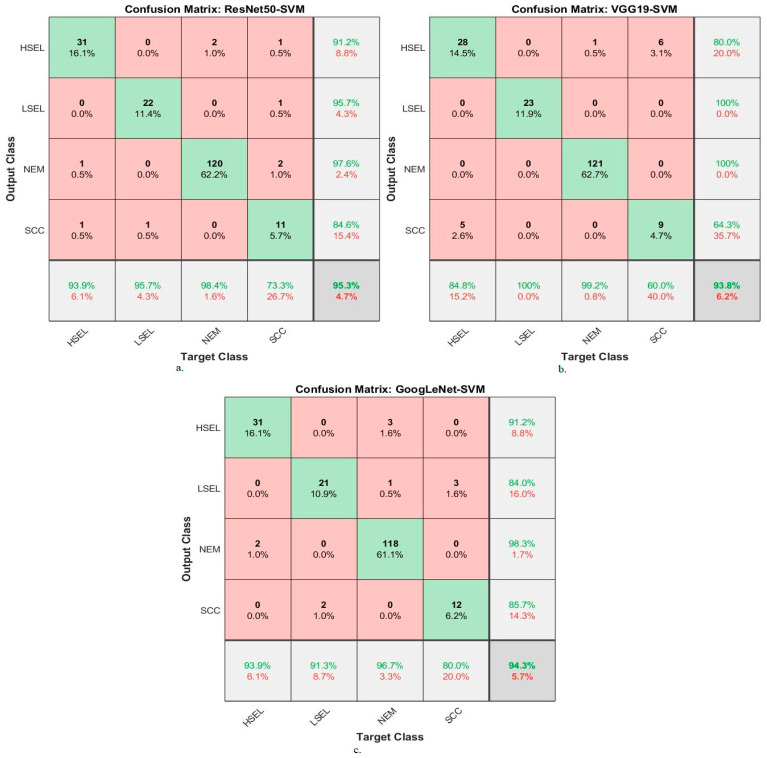
Confusion matrix for display performance results of DL with SVM based on ACA algorithm for analysis, and WSI image of a CESC dataset for the diagnosis and discrimination of cervical cancer.

The hybrid methodologies of DL-SVM produce confusion matrices that measure the performance of the hybrid methods for early diagnosis of cervical cancer and differentiation between stages of its development. Figure 11 displays the confusion matrix for the ResNet50-SVM, VGG19-SVM, and GoogLeNet-SVM models for analyzing WSI images for cervical cancer detection. The figure shows the accuracy reached by the ResNet50-SVM system for each class: 93.9% for HSEL, 95.7% for LSEL, 98.4% for NEM, and 73.3% for SCC. For the VGG19-SVM system, the accuracy for each class was achieved as follows: 84.8% for HSEL, 100% for LSEL, 99.2% for NEM, and 60% for SCC. The GoogLeNet-SVM system resulted in accuracy for each class as follows: 93.9% for HSEL, 91.3% for LSEL, 96.7% for NEM, and 80% for SCC.

### 3.7. Results of Hybrid Systems Based on CNN Combined Features

The section summarizes the performance of the hybrid methodology by RF and SVM algorithms based on fused DL models (ResNet50, VGG19, and GoogLeNet) for analyzing WSI images of cervical cancer. The methodology consists of two blocks, DL models that extract the deep features and then select essential features by PCA. Then, the features of the DL models are combined as follows: ResNet50-VGG19, VGG19-GoogLeNet, and ResNet50-GoogLeNet. The second block is the RF and SVM algorithms, which receive the combined features then split them 80% for training and validation while isolating 20% for testing the systems. Fusion-DL-RF and Fusion-DL-SVM hybrid methods have superior capabilities for analyzing WSI images of cervical cancer and differentiating between stages of progression with high efficiency.

**Figure 12:**
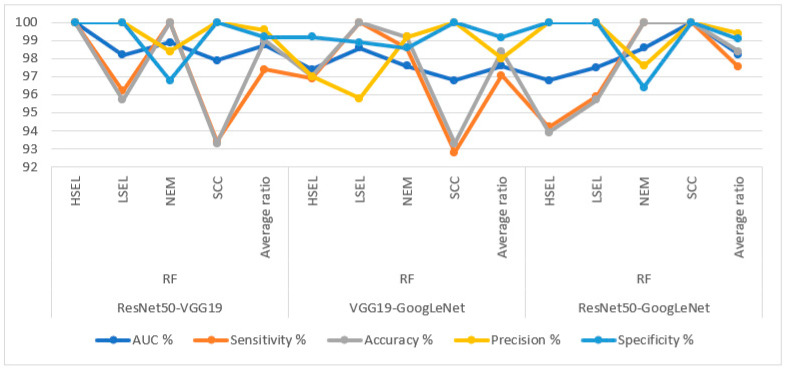
Display of performance results of RF method based on fusion features DL for analysis, and WSI image of a CESC dataset for the diagnosis and discrimination of cervical cancer.

Table 7 and Figure 12 summarize the performance of hybrid methodologies of RF algorithms based on fused DL models for analyzing WSI images for diagnosing and differentiating stages of cervical cancer development. ResNet50-VGG19 with RF reached an AUC of 98.75%, a sensitivity of 97.4%, an accuracy of 99%, a precision of 99.6%, and a specificity of 99.2%. The performance of the VGG19-GoogLeNet with RF reached an AUC of 97.6%, a sensitivity of 97.08%, an accuracy of 98.4%, a precision of 98%, and a specificity of 99.18%. In contrast, ResNet50-GoogLeNet with RF reached an AUC of 98.23%, a sensitivity of 97.53%, an accuracy of 98.5%, a precision of 99.4%, and a specificity of 99.1%.

**Figure 13:**
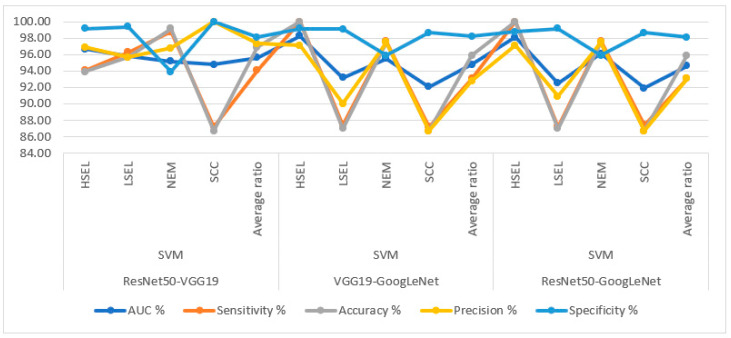
Display of performance results of SVM method based on fusion features DL for analysis, and WSI image of a CESC dataset for the diagnosis and discrimination of cervical cancer.

Table 8 and Figure 13 summarize the performance of hybrid methodologies of SVM algorithms based on fused DL models for analyzing WSI images for diagnosing and differentiating stages of cervical cancer development. ResNet50-VGG19 with SVM reached an AUC of 95.63%, a sensitivity of 94.1%, an accuracy of 96.9%, a precision of 97.35%, and a specificity of 98.13%. The performance of the VGG19-GoogLeNet with SVM reached an AUC of 94.78%, a sensitivity of 93.05%, an accuracy of 95.9%, a precision of 92.83%, and a specificity of 98.23%. In contrast, ResNet50-GoogLeNet with SVM reached an AUC of 94.65%, a sensitivity of 93.08%, an accuracy of 95.9%, a precision of 93.05%, and a specificity of 98.15%.

**Figure 14:**
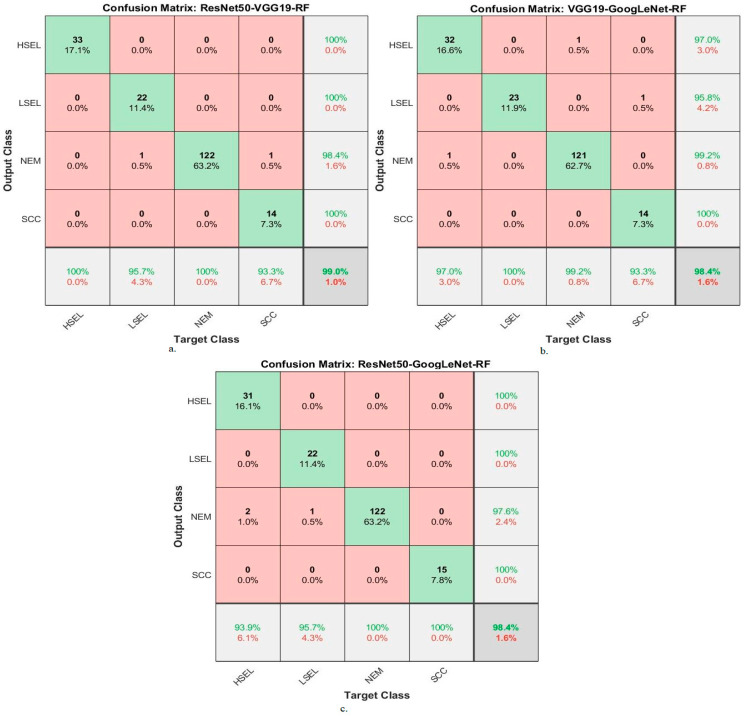
Confusion matrix for display performance results of RF method based on fusion features DL for analysis, and WSI image of a CESC dataset for the diagnosis and discrimination of cervical cancer.

Hybrid methodologies for RF with fused DL features produce confusion matrices that measure the performance of hybrid methods for early diagnosis and differentiation of cervical cancer stages. Figure 14 displays the confusion matrix for the ResNet50-VGG19-RF, VGG19-GoogLeNet-RF, and ResNet50-GoogLeNet-RF models for analyzing WSI images for cervical cancer detection. The figure shows the accuracy reached by the ResNet50-VGG19-RF system for each class: 100% for HSEL, 95.7% for LSEL, 100% for NEM, and 93.3% for SCC. Meanwhile, for the VGG19-GoogLeNet-RF system, the accuracy for each class was achieved as follows: 97% for HSEL, 100% for LSEL, 99.2% for NEM, and 93.3% for SCC. The ResNet50-GoogLeNet-RF system resulted in accuracy for each class as follows: 93.9% for HSEL, 95.7% for LSEL, 100% for NEM, and 100% for SCC.

**Figure 15:**
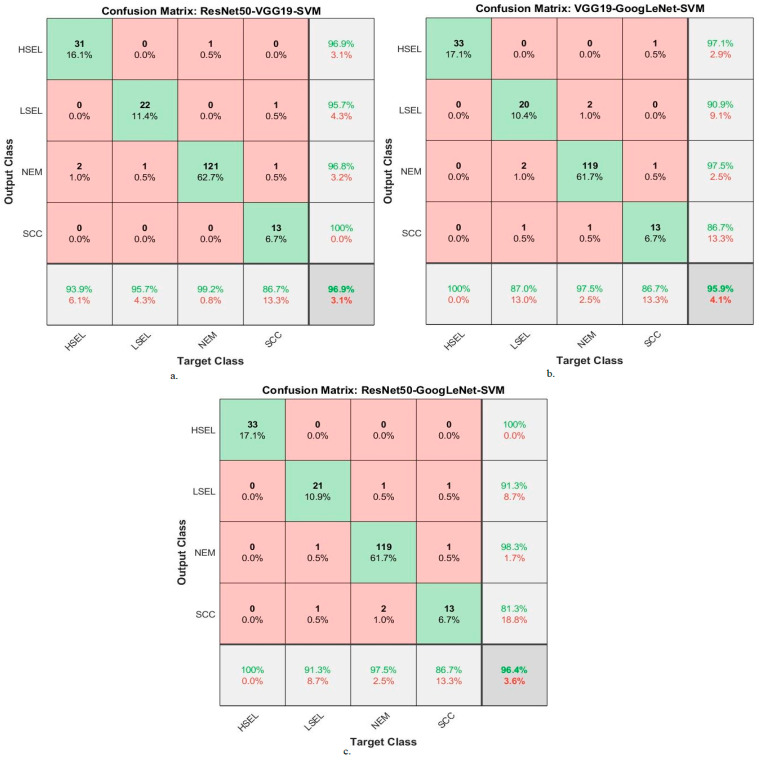
Confusion matrix for display performance results of SVM method based on fusion features DL for analysis, and WSI image of a CESC dataset for the diagnosis and discrimination of cervical cancer.

Hybrid methodologies for SVM with fused DL features produce confusion matrices that measure the performance of hybrid methods for early diagnosis and differentiation of cervical cancer stages. Figure 15 displays the confusion matrix for the ResNet50-VGG19-SVM, VGG19-GoogLeNet-SVM, and ResNet50-GoogLeNet-SVM models for analyzing WSI images for cervical cancer detection. The figure shows the accuracy reached by the ResNet50-VGG19-SVM system for each class: 93.9% for HSEL, 95.7% for LSEL, 99.2% for NEM, and 86.7% for SCC. Meanwhile, for the VGG19-GoogLeNet-SVM system, the accuracy for each class was achieved as follows: 100% for HSEL, 87% for LSEL, 97.5% for NEM, and 86.7% for SCC. The ResNet50-GoogLeNet-SVM system resulted in accuracy for each class as follows: 100% for HSEL, 91.3% for LSEL, 97.5% for NEM, and 86.7% for SCC.

## 4. Discussion of the Performance

Cervical cancer is one of the deadliest types of ailments for women. HPV is one of the main causes of cervical cancer. Early diagnosis of cervical cancer is the only means to receive appropriate treatment and recover. The similarity between the vital characteristics of the types of cervical cancer or the stages of cancer development makes it difficult for doctors to distinguish the stages. Thus, artificial intelligence techniques solve the shortcomings of manual diagnosis. Many researchers discussed various techniques and algorithms and their findings. It is noted from previous studies that there are shortcomings in terms of pathological accuracy. Therefore, due to the similarity of clinical characteristics between cancer stages, this study aimed to extract features from several DL models and combine them.

In this study, WSI images for diagnosing squamous cell carcinoma of the cervix were analyzed by several approaches. The first approach to analyze WSI images for cervical cancer diagnosis is through pre-trained ResNet50, VGG19, and GoogLeNet models. The ResNet50, VGG19, and GoogLeNet models achieved an accuracy of 91.7%, 92.2%, and 90.2%, respectively.

The second approach to analyze WSI images for cervical cancer diagnosis through ResNet50, VGG19, and GoogLeNet models was based on the ACA algorithm to segment and isolate regions of interest from non-significant regions. The second approach notes an improvement in the performance of ResNet50, VGG19, and GoogLeNet models thanks to the segmentation algorithm before feeding images to DL models. ResNet50, VGG19, and GoogLeNet models based on the ACA algorithm achieved an accuracy of 94.8%, 95.9%, and 92.7%.

The third approach is based on a hybrid of DL models and RF and SVM algorithms. In this technique, ResNet50, VGG19, and GoogLeNet models were fed after applying the ACA algorithm, and the images were analyzed to extract the high-dimensional features. The PCA method receives high-dimensional features to select representative features and eliminate non-significant and redundant features. Finally, the RF and SVM algorithm receives representative features to classify each image into the appropriate class with high accuracy. It is noted that the results of the RF algorithm based on the characteristics of DL demonstrated greater improvement than the first and second approaches. The ResNet50-RF, VGG19-RF, and GoogLeNet-RF approaches achieved an accuracy of 97.4%, 96.9%, and 97.9%, respectively.

The fourth approach is based on analyzing WSI images for cervical cancer diagnosis by RF and SVM algorithms based on integrating the features of DL models. In this approach, WSI image cells are segmented and fed into ResNet50, VGG19, and GoogLeNet models, WSI image cells are analyzed, and high-level features are extracted. After that, the PCA method was applied to eliminate the repeated features and save the most important features. The features of the low-dimensional DL models are combined: ResNet50-VGG19, VGG19-GoogLeNet, and ResNet50-GoogLeNet. Finally, the RF and SVM algorithms receive the DL-fused features. Experiments with this approach have proven high performance compared to previous approaches thanks to the combination of the features of DL models. The RF algorithm with the features of the models ResNet50-VGG19, VGG19-GoogLeNet, and ResNet50-GoogLeNet reached an accuracy of 99%, 98.4%, and 98.5%, respectively.

The reason behind the improvement in results across the approaches lies in the combination of different techniques and algorithms. The first approach leverages pre-trained DL models to directly classify the WSI images, but without any specific preprocessing. The second approach introduces the ACA algorithm for segmentation, isolates the regions of interest, and reduces noise, which enhances the DL models’ performance. The third approach combines DL models with RF and SVM algorithms, allowing for better feature representation and classification. Finally, the fourth approach integrates the features from different DL models, further refining the representation of the images and achieving the highest accuracy. Each subsequent approach builds upon the strengths and limitations of the previous ones, resulting in incremental improvements in performance.

In our study, a system for cervical cancer diagnosis based on analyzing whole slide imaging (WSI) images was proposed. We compare our approach with several previous studies that employed different methods and achieved varying results. Here, we summarize the previous studies and their outcomes. Kavitha et al. [18] used Dynamic Fuzzy Histogram Equalization for image enhancement and fuzzy c-mean for the region of interest identification. Ant colony optimization, CNN, and ANN were used for trait selection and classification. Badiea et al. [19] applied a hybrid method using CNN and SVM for WSI image analysis. GoogLeNet features combined with SVM achieved an accuracy of 96.8%. Hiam et al. [20] integrated DL features with Shuffle Net for cervical cancer classification. PCA and CCA were used for feature extraction, and SVM, RF, and ANN achieved accuracies of 91.1%, 94.7%, and 94.9%, respectively. Jesse et al. [21] used a decision tree algorithm and RFE for feature selection in cervical cancer risk factor analysis. The decision tree achieved an accuracy of 95.29%. Odai et al. [22] presented a system for predicting cervical cancer using GM, PCA, and MLP. RF achieved an accuracy of 92.16%, a specificity of 85.57%, and a precision of 87.22%. Mingmei et al. [23] applied unsupervised ML for analyzing cervical cytology images. Their method achieved a sensitivity of 81.8% and specificity of 86.5%. Park et al. [24] used DL and ML methods for cervical cancer detection. XGB and SVM achieved AUC values of 82% and 84%, respectively. Yoon et al. [25] employed a CNN-based method for cervical cancer detection using acetowhite images. The accuracy of their method was reported as 81.31%. Naif et al. [26] developed ML methods based on image optimization and feature selection using PMS. No specific accuracy results were provided. Phasit et al. [27] developed an RF-based prediction model for cervical cancer using iPMI-Econ and iPMI-Power features. Their method achieved a superiority of 86.2%, sensitivity of 60%, and an AUC of 90.5%. Madhura et al. [28] applied a hybrid method combining machine learning and fuzzy min–max neural networks. Their approach achieved an accuracy of 95.33% using ResNet-50. Deborah et al. [29] performed feature analysis and hierarchical classification of cervical cells using ML algorithms. RF achieved an accuracy of 95.34%, a precision of 84.31%, and a sensitivity of 84%. Mohammed et al. [30] used DL networks for analyzing WSI images of cervical cells. After image improvement, their systems showed better performance, but specific accuracy results were not provided. Débora et al. [31] used DL models, specifically XceptionNet, for cervical cancer detection. The accuracy, sensitivity, and specificity achieved were 93%, 80%, and 80%, respectively. Shervan et al. [32] employed deep networks (MLP, KNN, and RF) for feature extraction and classification. Accuracy results were reported as 96.53%, 94.43%, and 95.51% for MLP, KNN, and RF, respectively. In our proposed system, we analyze WSI images for cervical cancer diagnosis using RF and SVM algorithms. We integrate features from DL models, specifically ResNet50, VGG19, and GoogLeNet. The features are combined using PCA to eliminate redundant features. The fused features from ResNet50-VGG19, VGG19-GoogLeNet, and ResNet50-GoogLeNet are then fed into RF and SVM algorithms. Our experiments showed that our approach outperformed previous methods. The RF algorithm achieved accuracies of 99%, 98.4%, and 98.5% with ResNet50-VGG19, VGG19-GoogLeNet, and ResNet50-GoogLeNet features, respectively. Overall, our proposed system demonstrates improved performance compared to previous studies by leveraging the combination of features from DL models and utilizing RF and SVM algorithms for classification.

We conclude that the segmentation of WSI images of cervical cancer cells by the ACA algorithm was effective in improving performance. The DL-RF and DL-SVM hybrid models perform better than the pre-trained DL models. We also conclude that the performance of the two RF and SVM algorithms based on the merged DL models is better than its performance with DL features without merging the feature vectors.

## 5. Conclusions

This work has developed several hybrid models based on the advantages fused between DL models. This work analyzes WSI images of cervical cancer cells and the multiple risk factors that lead to their development. Effective methodologies have been developed to distinguish between types of cervical cancer and its stages of development by identifying gaps in previous studies. WSI images of cervical cancer are improved to show borderline contrast of the cells to be analyzed. To only analyze the important cervical cells, the ACA algorithm was applied for extracting only the important regions for further analysis in the following stages. Then, two approaches were applied to analyze the important image cells and classify them as follows. The first approach to analyze WSI image cells is a hybrid technique between RF and SVM algorithms based on the features of ResNet50, VGG19, and GoogLeNet models. The second approach to analyze WSI image cells is a hybrid technique between RF and SVM algorithms based on fused features between DL models (ResNet50-VGG19, VGG19-GoogLeNet, and ResNet50-GoogLeNet). We conclude that the performance of the RF and SVM algorithms with fused features of DL models is better than their performance with DL features without fusion. The RF algorithm with fused features of the ResNet50-VGG19 models achieved an AUC of 98.75%, a sensitivity of 97.4%, an accuracy of 99%, a precision of 99.6%, and a specificity of 99.2%.

## Figures and Tables

**Table 1:** Splitting the CESC datasets of cervical.

Phase	80% (80:20)		Testing 20%
Classes	Training (80%)	Validation (20%)	
High-squamous intra-epithelial lesion	104	26	33
Squamous intra-epithelial lesion	72	18	23
Negative for intra-epithelial malignancy	392	98	122
Squamous cell carcinoma	47	12	15

**Table 2:** Balancing WSI image of CESC dataset of cervical cancer.

Phase	Training Dataset			
Classes	HSEL	LSEL	NEM	SCC
Bef-augm	104	72	392	47
Aft-augm	**1144**	**1152**	**1176**	**1175**

**Table 3:** Performance results of DL models for analysis, and WSI image of a CESC dataset for the diagnosis and discrimination of cervical cancer.

Models	Lesion Type	AUC %	Sensitivity %	Accuracy %	Precision %	Specificity %
ResNet50	HSEL	90.20	84.80	84.80	80.00	96.20
	LSEL	96.20	95.90	95.70	88.00	98.10
	NEM	95.10	98.30	97.50	96.70	93.80
	SCC	64.60	53.10	53.30	80.00	98.90
	**Average ratio**	**86.53**	**83.03**	**91.70**	**86.18**	**96.75**
VGG19	HSEL	95.80	97.20	97.00	84.20	95.80
	LSEL	88.30	82.80	82.60	86.40	97.90
	NEM	93.50	96.90	96.70	95.90	93.30
	SCC	66.80	60.30	60.00	90.00	99.10
	**Average ratio**	**86.10**	**84.30**	**92.20**	**89.13**	**96.53**
GoogLeNet	HSEL	89.20	73.20	72.70	85.70	97.20
	LSEL	93.40	96.10	95.70	81.50	96.80
	NEM	96.20	95.80	95.90	95.90	93.10
	SCC	86.70	73.20	73.30	68.80	96.20
	**Average ratio**	**91.38**	**84.58**	**90.20**	**82.98**	**95.83**

**Table 4:** Performance results of DL models based on ACA algorithm for analysis; WSI image of a CESC dataset for the diagnosis and discrimination of cervical cancer.

Models	Lesion Type	AUC %	Sensitivity %	Accuracy %	Precision %	Specificity %
ResNet50	HSEL	94.20	93.80	93.90	96.90	98.80
	LSEL	95.50	91.20	91.30	91.30	99.10
	NEM	96.70	98.10	98.40	97.60	96.20
	SCC	89.20	72.90	73.30	73.30	97.70
	**Average ratio**	**93.90**	**89.00**	**94.80**	**89.78**	**97.95**
VGG19	HSEL	93.60	91.20	90.90	90.90	97.90
	LSEL	100	100	100	100	100
	NEM	98.80	100	100	98.40	96.80
	SCC	82.10	66.90	66.70	76.90	98.20
	**Average ratio**	**93.63**	**89.53**	**95.90**	**91.55**	**98.23**
GoogLeNet	HSEL	95.20	94.20	93.90	88.60	97.10
	LSEL	86.90	78.20	78.30	81.80	98.20
	NEM	97.40	98.10	97.50	96.00	92.90
	SCC	82.70	72.80	73.30	91.70	99.20
	**Average ratio**	**90.55**	**85.83**	**92.70**	**89.53**	**96.85**

**Table 5:** Performance results of DL with RF based on ACA algorithm for analysis, and WSI image of a CESC dataset for the diagnosis and discrimination of cervical cancer.

Models	Lesion Type	AUC %	Sensitivity %	Accuracy %	Precision %	Specificity %
ResNet50-RF	HSEL	96.10	94.30	93.90	93.90	98.70
	LSEL	97.80	96.20	95.70	95.70	99.10
	NEM	98.90	99.10	99.20	98.40	97.20
	SCC	96.80	92.70	93.30	100	100
	**Average ratio**	**97.40**	**95.58**	**97.40**	**97.00**	**98.75**
VGG19-RF	HSEL	96.50	97.40	97.00	94.10	99.10
	LSEL	98.70	100	100	95.80	98.90
	NEM	97.40	96.60	97	99.20	99.20
	SCC	94.90	93.20	93.30	87.50	98.70
	**Average ratio**	**96.88**	**96.80**	**96.90**	**94.15**	**98.98**
GoogLeNet-RF	HSEL	96.80	97.20	97.00	97.00	99.30
	LSEL	97.20	96.30	95.70	100	100
	NEM	98.10	98.80	99.20	99.20	98.80
	SCC	94.90	92.60	93.30	87.50	98.70
	**Average ratio**	**96.75**	**96.23**	**97.90**	**95.93**	**99.20**

**Table 6:** Performance results of DL with SVM based on ACA algorithm for analysis, and WSI image of a CESC dataset for the diagnosis and discrimination of cervical cancer.

Models	Lesion Type	AUC %	Sensitivity %	Accuracy %	Precision %	Specificity %
ResNet50-SVM	HSEL	93.80	94.20	93.90	91.20	98.20
	LSEL	96.60	95.80	95.70	95.70	99.10
	NEM	98.20	97.80	98.40	97.60	96.30
	SCC	80.50	73.90	73.30	84.60	99.40
	**Average ratio**	**92.28**	**90.43**	**95.30**	**92.28**	**98.25**
VGG19-SVM	HSEL	90.40	85.20	84.80	80.00	96.40
	LSEL	100	100	100	100	100
	NEM	99.50	98.80	99.20	100	100
	SCC	78.50	60.30	80.00	64.30	96.80
	**Average ratio**	**92.10**	**86.08**	**93.80**	**86.1**	**98.30**
GoogLeNet-SVM	HSEL	95.40	94.20	93.90	91.20	98.10
	LSEL	92.10	90.80	91.30	84.00	97.70
	NEM	96.70	97.20	96.70	98.30	96.80
	SCC	85.60	80.20	80.00	85.70	99.20
	**Average ratio**	**92.45**	**90.60**	**94.30**	**89.80**	**97.95**

**Table 7:** Performance results of RF method based on fusion features DL for analysis, and WSI image of a CESC dataset for the diagnosis and discrimination of cervical cancer.

Fusion Features	Classifier	Lesion Type	AUC %	Sensitivity %	Accuracy %	Precision %	Specificity %
ResNet50-VGG19	RF	HSEL	100	100	100	100	100
		LSEL	98.20	96.20	95.70	100	100
		NEM	98.90	100	100	98.40	96.80
		SCC	97.90	93.40	93.30	100	100
		**Average ratio**	**98.75**	**97.40**	**99.00**	**99.60**	**99.20**
VGG19-GoogLeNet	RF	HSEL	97.40	96.90	97.00	97.00	99.20
		LSEL	98.60	100	100	95.80	98.90
		NEM	97.60	98.60	99.20	99.20	98.60
		SCC	96.80	92.80	93.30	100	100
		**Average ratio**	**97.60**	**97.08**	**98.40**	**98.00**	**99.18**
ResNet50-GoogLeNet	RF	HSEL	96.80	94.20	93.90	100	100
		LSEL	97.50	95.90	95.70	100	100
		NEM	98.60	100	100	97.60	96.40
		SCC	100	100	100	100	100
		**Average ratio**	**98.23**	**97.53**	**98.50**	**99.40**	**99.10**

**Table 8:** Performance results of SVM method based on fusion features DL for analysis WSI image of a CESC dataset for the diagnosis and discrimination of cervical cancer.

Fusion Features	Classifier	Lesion Type	AUC %	Sensitivity %	Accuracy %	Precision %	Specificity %
ResNet50-VGG19	SVM	HSEL	96.70	94.10	93.90	96.90	99.20
		LSEL	95.80	96.30	95.70	95.70	99.40
		NEM	95.20	98.80	99.20	96.80	93.90
		SCC	94.80	87.20	86.70	100	100
		**Average ratio**	**95.63**	**94.10**	**96.90**	**97.35**	**98.13**
VGG19-GoogLeNet	SVM	HSEL	98.30	100	100	97.10	99.20
		LSEL	93.20	87.40	87.00	90.00	99.10
		NEM	95.50	97.60	97.50	97.50	95.90
		SCC	92.10	87.20	86.70	86.70	98.70
		**Average ratio**	**94.78**	**93.05**	**95.90**	**92.83**	**98.23**
ResNet50-GoogLeNet	SVM	HSEL	98.10	100	100	97.10	98.80
		LSEL	92.50	87.20	87.00	90.90	99.20
		NEM	96.10	97.70	97.50	97.50	95.90
		SCC	91.90	87.40	86.70	86.70	98.70
		**Average ratio**	**94.65**	**93.08**	**95.90**	**93.05**	**98.15**

## Data Availability

X Data that support the systems proposed in this work were collected from a public dataset available online to the public at the following link: https://data.mendeley.com/datasets/zddtpgzv63/4 (accessed on 13 October 2022).
